# Diaqua­(5,5,7,12,12,14-hexa­methyl-1,4,8,11-tetra­azacyclo­tetra­deca­ne)nickel(II) tetra­cyanidonickelate(II)

**DOI:** 10.1107/S1600536809033820

**Published:** 2009-08-29

**Authors:** Qian Zhang, Xiao-Ping Shen, Hu Zhou

**Affiliations:** a School of Chemistry and Chemical Engineering, Jiangsu University, Zhenjiang 212013, People’s Republic of China

## Abstract

In the title complex, [Ni(C_16_H_36_N_4_)(H_2_O)_2_][Ni(CN)_4_], the [Ni(teta)(H_2_O)_2_]^2+^ cations (teta = 5,5,7,12,12,14-hexa­methyl-1,4,8,11-tetra­azacyclo­tetra­deca­ne) and [Ni(CN)_4_]^2−^ anions are arranged in an alternating fashion through electrostatic and N—H⋯N and O—H⋯N hydrogen-bonding inter­actions, forming a two-dimensional layered structure. Adjacent layers are linked through weak van der Waals inter­actions, resulting in a three-dimensional supra­molecular network.

## Related literature

For background to cyanide-bridged complexes, see: Lescouëzec *et al.* (2005 ▶); Liu *et al.* (2008 ▶); Xu *et al.* (2009 ▶). For related structures, see: Jiang *et al.* (2005 ▶, 2007 ▶); Ni *et al.* (2008 ▶); Yamada & Iwasaki (1969 ▶).
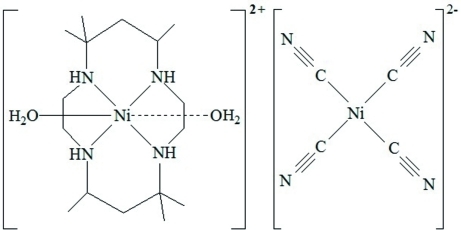


## Experimental

### 

#### Crystal data

[Ni(C_16_H_36_N_4_)(H_2_O)_2_][Ni(CN)_4_]
*M* *_r_* = 542.02
Monoclinic, 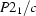

*a* = 8.065 (8) Å
*b* = 13.255 (12) Å
*c* = 13.559 (10) Å
β = 116.59 (4)°
*V* = 1296 (2) Å^3^
*Z* = 2
Mo *K*α radiation
μ = 1.48 mm^−1^
*T* = 173 K
0.58 × 0.16 × 0.12 mm

#### Data collection

Bruker SMART APEX diffractometer
Absorption correction: multi-scan (*SADABS*; Bruker, 2004 ▶) *T*_min_ = 0.808, *T*_max_ = 0.888
9778 measured reflections
2530 independent reflections
1576 reflections with *I* > 2σ(*I*)
*R*_int_ = 0.047

#### Refinement

*R*[*F* ^2^ > 2σ(*F* ^2^)] = 0.032
*wR*(*F* ^2^) = 0.093
*S* = 1.01
2530 reflections
163 parameters
2 restraints
H atoms treated by a mixture of independent and constrained refinement
Δρ_max_ = 0.73 e Å^−3^
Δρ_min_ = −0.51 e Å^−3^

### 

Data collection: *SMART* (Bruker, 2004 ▶); cell refinement: *SAINT* (Bruker, 2004 ▶); data reduction: *SAINT*; program(s) used to solve structure: *SHELXS97* (Sheldrick, 2008 ▶); program(s) used to refine structure: *SHELXL97* (Sheldrick, 2008 ▶); molecular graphics: *SHELXTL* (Sheldrick, 2008 ▶) and *DIAMOND* (Brandenburg, 2006 ▶); software used to prepare material for publication: *SHELXL97*.

## Supplementary Material

Crystal structure: contains datablocks I, global. DOI: 10.1107/S1600536809033820/at2863sup1.cif

Structure factors: contains datablocks I. DOI: 10.1107/S1600536809033820/at2863Isup2.hkl

Additional supplementary materials:  crystallographic information; 3D view; checkCIF report

## Figures and Tables

**Table 1:** Hydrogen-bond geometry (Å, °)

*D*—H⋯*A*	*D*—H	H⋯*A*	*D*⋯*A*	*D*—H⋯*A*
N4—H4⋯N1^i^	0.81 (3)	2.46 (3)	3.250 (4)	164 (3)
N3—H3⋯N2	0.88 (3)	2.34 (3)	3.201 (4)	167 (3)
O1—H1*B*⋯N2	0.830 (10)	1.964 (11)	2.789 (4)	172 (3)
O1—H1*A*⋯N1^ii^	0.835 (10)	1.939 (11)	2.775 (4)	179 (3)

Symmetry codes: (i) 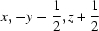; (ii) 
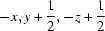
.

**Fig. 1.**
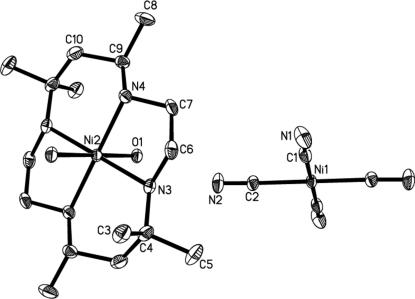
ORTEP view of the title complex. Displacement ellipsoids are drawn at the 30% probability level. Hydrogen atoms have been omitted for clarity.

**Fig. 2.**
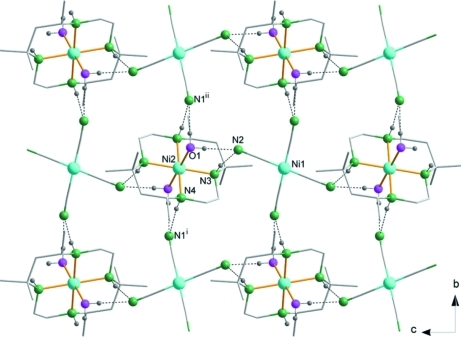
Projection of the title complex viewed from the a-axis, showing the two-dimensional structure. Hydrogen bonds are shown as dashed lines. Symmetry codes: (i) x, -y-0.5, z+0.5; (ii) -x, y+0.5, -z+0.5.

**Fig. 3.**
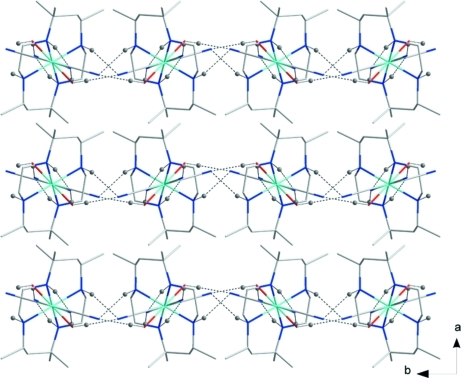
The three-dimensional supramolecular network of the title complex.

## supplementary crystallographic information

### Comment

In the past decades, there has been a continuous interest in the utilization of cyano-containing building blocks for constructing either ion-paired or cyano-bridged assemblies due to their potential applications and intriguing architectures (Lescouëzec *et al.*, 2005; Liu *et al.*, 2008; Xu *et al.*, 2009). It has been found that cyano-bridged bimetallic assemblies, derived from tailored cyanometalate entities [*ML_p_*(CN)*_q_*]^n-^ (*L* = polydentate ligand) and unsaturated coordinated complex [*M*'(*L*)]^m+^, possess extraordinarily excellent magnetic properties such as SMM (single molecular magnets) and SCM (single chain magnets). Recently, we had expected to obtain such low-dimensional system using [Cr(salen)(CN)_2_]^-^ (Yamada *et al.*, 1969; Ni *et al.*, 2008) and [Ni(teta)]^2+^ as the building blocks. However, an unexpected tetracyanonickel(II)-based complex of [Ni(teta)(H_2_O)_2_][Ni(CN)_4_] instead of any [Cr(salen)(CN)_2_]^-^-based complex was obtained. So far, Jiang *et al.* (Jiang *et al.*, 2005; 2007) have reported several complexes based on the direct assembly of [Ni(CN)_4_]^2-^ and [Ni(teta)]^2+^ building blocks, and they found that all these complexes showed cyano-bridged structures. In contrast to these reported complexes, the title complex of [Ni(teta)(H_2_O)_2_][Ni(CN)_4_] is ion-paired and its crystal structure is reported here.

The title complex consists of [Ni(teta)(H_2_O)_2_]^2+^ cation and [Ni(CN)_4_]^2-^ anion (Fig. 1). In [Ni(teta)(H_2_O)_2_]^2+^ cation, the Ni^II^ ion assumes a distorted octahedral coordination geometry, in which the equatorial sites are occupied by four nitrogen atoms of the macrocyclic ligand teta with the Ni(2)—N bond distances of 2.067 (3) and 2.100 (3) Å, while the axial positions are occupied by two oxygen atoms of water molecules with Ni(2)—O distance of 2.183 (2) Å. As usual, [Ni(CN)_4_]^2-^ anion exhibits a square planar structure, where all four cyano groups are terminal ones, with Ni(1)—C(1) and Ni(1)—C(2) distances of 1.862 (3) and 1.869 (3) Å, respectively. The Ni(1)—C—N bonds deviate slightly from linearity with the bond angles 177.2 (3) and 178.1 (3)°. [Ni(teta)(H_2_O)_2_]^2+^ and [Ni(CN)_4_]^2-^ are arranged in an alternating fashion, forming a two-dimensional layered structure through electrostatic and hydrogen bonding interactions (Fig. 2). Furthermore, adjacent layers are linked through weak van der Waals interactions, resulting in a three-dimensional supramolecular network (Fig. 3).

### Experimental

A solution of Ni(teta)(ClO_4_)_2_ (0.05 mmol) in DMF (10 ml) was added to a solution of K[Cr(salen)(CN)_2_].H_2_O (0.05 mmol) in MeOH/H_2_O (1/1(V/V),10 ml) mixture. The resulting solution was filtrated and the filtrate was left to allow slow evaporation in dark at room temperature. Pink prism crystals of the title complex were obtained after two weeks, washed with MeOH and H_2_O, respectively, and dried in air. Anal. Calc. for C_20_H_40_Ni_2_N_8_O_2_: C, 44.32; H, 7.44; N, 20.68; Ni, 21.66%. Found: C, 44.28; H, 7.49; N, 20.71; Ni, 21.52%.

### Refinement

All non-H atoms were refined anisotropically. The C(H) atoms of the teta ligands were placed incalculated position [C-H = 0.99 Å or 0.98 Å] and refined using a riding model, with *U*_iso_(H) = 1.2*U*_eq_(C) or *U*_iso_(H) = 1.5*U*_eq_(C). The N(H) atoms were located from the difference Fourier maps, and refined as riding with *U*_iso_(H) = 1.2*U*_eq_(N). The O(H) atoms of the coordinated water molecules were located in a difference Fourier map and refined as riding [O-H = 0.84 Å], with *U*_iso_(H) = 1.5*U*_eq_(O).

### Crystal data

**Table:**

[Ni(C_16_H_36_N_4_)(H_2_O)_2_][Ni(CN)_4_]	*F*(000) = 576
*M**_r_* = 542.02	*D*_x_ = 1.389 Mg m^−^^3^
Monoclinic, *P*2_1_/*c*	Mo *K*α radiation, λ = 0.71073 Å
Hall symbol: -P 2ybc	Cell parameters from 4392 reflections
*a* = 8.065 (8) Å	θ = 2.3–26.0°
*b* = 13.255 (12) Å	µ = 1.48 mm^−^^1^
*c* = 13.559 (10) Å	*T* = 173 K
β = 116.59 (4)°	Prism, pink
*V* = 1296 (2) Å^3^	0.58 × 0.16 × 0.12 mm
*Z* = 2	

### Data collection

**Table:**

Bruker SMART APEX diffractometer	1576 reflections with *I* > 2σ(*I*)
Radiation source: fine-focus sealed tube	*R*_int_ = 0.047
φ and ω scans	θ_max_ = 26.0°, θ_min_ = 2.3°
Absorption correction: multi-scan (*SADABS*; Bruker, 2004)	*h* = −9→9
*T*_min_ = 0.808, *T*_max_ = 0.888	*k* = −16→15
9778 measured reflections	*l* = −16→16
2530 independent reflections	

### Refinement

**Table:**

Refinement on *F*^2^	Primary atom site location: structure-invariant direct methods
Least-squares matrix: full	Secondary atom site location: difference Fourier map
*R*[*F*^2^ > 2σ(*F*^2^)] = 0.032	Hydrogen site location: inferred from neighbouring sites
*wR*(*F*^2^) = 0.093	H atoms treated by a mixture of independent and constrained refinement
*S* = 1.01	*w* = 1/[σ^2^(*F*_o_^2^) + (0.0423*P*)^2^ + 0.2883*P*] where *P* = (*F*_o_^2^ + 2*F*_c_^2^)/3
2530 reflections	(Δ/σ)_max_ < 0.001
163 parameters	Δρ_max_ = 0.73 e Å^−^^3^
2 restraints	Δρ_min_ = −0.51 e Å^−^^3^

### Special details

**Table:**

Geometry. All esds (except the esd in the dihedral angle between two l.s. planes) are estimated using the full covariance matrix. The cell esds are taken into account individually in the estimation of esds in distances, angles and torsion angles; correlations between esds in cell parameters are only used when they are defined by crystal symmetry. An approximate (isotropic) treatment of cell esds is used for estimating esds involving l.s. planes.
Refinement. Refinement of F^2^ against ALL reflections. The weighted R-factor wR and goodness of fit S are based on F^2^, conventional R-factors R are based on F, with F set to zero for negative F^2^. The threshold expression of F^2^ > 2sigma(F^2^) is used only for calculating R-factors(gt) etc. and is not relevant to the choice of reflections for refinement. R-factors based on F^2^ are statistically about twice as large as those based on F, and R- factors based on ALL data will be even larger.

### Fractional atomic coordinates and isotropic or equivalent isotropic displacement parameters (Å^2^)

**Table:**

	*x*	*y*	*z*	*U*_iso_*/*U*_eq_	
Ni1	0.0000	0.0000	0.0000	0.02563 (16)	
Ni2	0.0000	0.0000	0.5000	0.02354 (15)	
O1	0.1715 (3)	0.08968 (15)	0.44654 (15)	0.0298 (5)	
H1A	0.153 (5)	0.1515 (9)	0.449 (3)	0.045*	
H1B	0.146 (4)	0.086 (2)	0.3803 (10)	0.045*	
N1	−0.1059 (5)	−0.2055 (2)	0.0474 (2)	0.0537 (8)	
N2	0.0522 (4)	0.0799 (2)	0.2194 (2)	0.0477 (7)	
N3	−0.1760 (3)	−0.02613 (18)	0.33256 (18)	0.0278 (6)	
H3	−0.120 (4)	0.012 (2)	0.303 (2)	0.033*	
N4	0.1362 (3)	−0.12904 (18)	0.49057 (19)	0.0282 (6)	
H4	0.097 (4)	−0.176 (2)	0.513 (2)	0.034*	
C1	−0.0637 (4)	−0.1267 (2)	0.0321 (2)	0.0358 (7)	
C2	0.0293 (4)	0.0502 (2)	0.1355 (2)	0.0321 (7)	
C3	−0.4969 (4)	−0.0695 (3)	0.3047 (2)	0.0441 (8)	
H3A	−0.5060	−0.1329	0.2655	0.066*	
H3B	−0.6212	−0.0410	0.2806	0.066*	
H3C	−0.4408	−0.0823	0.3842	0.066*	
C4	−0.3767 (4)	0.0051 (2)	0.2793 (2)	0.0368 (8)	
C5	−0.4462 (5)	0.0123 (3)	0.1536 (3)	0.0538 (10)	
H5A	−0.3779	0.0654	0.1371	0.081*	
H5B	−0.5788	0.0283	0.1181	0.081*	
H5C	−0.4261	−0.0524	0.1256	0.081*	
C6	−0.1359 (4)	−0.1309 (2)	0.3117 (2)	0.0368 (8)	
H6A	−0.1808	−0.1416	0.2316	0.044*	
H6B	−0.2009	−0.1791	0.3382	0.044*	
C7	0.0699 (4)	−0.1490 (2)	0.3712 (2)	0.0348 (7)	
H7A	0.0977	−0.2197	0.3601	0.042*	
H7B	0.1344	−0.1039	0.3413	0.042*	
C8	0.4289 (5)	−0.2269 (3)	0.5442 (3)	0.0530 (10)	
H8A	0.3772	−0.2838	0.5674	0.080*	
H8B	0.5634	−0.2242	0.5901	0.080*	
H8C	0.4023	−0.2355	0.4668	0.080*	
C9	0.3411 (4)	−0.1285 (2)	0.5574 (2)	0.0348 (7)	
H9	0.3932	−0.0713	0.5319	0.042*	
C10	0.3891 (4)	−0.1115 (2)	0.6794 (2)	0.0412 (8)	
H10A	0.3070	−0.1558	0.6969	0.049*	
H10B	0.5175	−0.1358	0.7238	0.049*	

### Atomic displacement parameters (Å^2^)

**Table:**

	*U*^11^	*U*^22^	*U*^33^	*U*^12^	*U*^13^	*U*^23^
Ni1	0.0380 (3)	0.0219 (3)	0.0229 (3)	−0.0001 (2)	0.0188 (2)	0.0007 (2)
Ni2	0.0290 (3)	0.0228 (3)	0.0216 (3)	0.0012 (2)	0.0138 (2)	0.0004 (2)
O1	0.0416 (12)	0.0258 (12)	0.0265 (10)	0.0003 (11)	0.0195 (9)	−0.0004 (10)
N1	0.093 (2)	0.0286 (16)	0.069 (2)	−0.0054 (17)	0.0625 (19)	−0.0004 (15)
N2	0.078 (2)	0.0434 (17)	0.0344 (14)	−0.0084 (16)	0.0364 (15)	−0.0043 (13)
N3	0.0293 (14)	0.0323 (15)	0.0234 (12)	−0.0030 (11)	0.0134 (11)	0.0006 (10)
N4	0.0342 (15)	0.0242 (14)	0.0339 (13)	0.0020 (12)	0.0221 (11)	0.0032 (11)
C1	0.052 (2)	0.0330 (19)	0.0348 (16)	0.0011 (16)	0.0302 (16)	−0.0012 (14)
C2	0.0469 (19)	0.0269 (18)	0.0293 (15)	0.0004 (15)	0.0232 (14)	0.0052 (13)
C3	0.0329 (18)	0.058 (2)	0.0374 (17)	−0.0086 (17)	0.0117 (14)	0.0059 (16)
C4	0.0325 (17)	0.046 (2)	0.0281 (15)	−0.0030 (16)	0.0100 (13)	0.0061 (14)
C5	0.048 (2)	0.076 (3)	0.0265 (16)	−0.0072 (19)	0.0068 (15)	0.0096 (17)
C6	0.052 (2)	0.0327 (19)	0.0296 (15)	−0.0100 (16)	0.0220 (15)	−0.0087 (14)
C7	0.049 (2)	0.0287 (18)	0.0381 (17)	0.0009 (15)	0.0300 (15)	−0.0054 (13)
C8	0.052 (2)	0.042 (2)	0.078 (3)	0.0205 (17)	0.040 (2)	0.0140 (18)
C9	0.0366 (18)	0.0316 (18)	0.0446 (17)	0.0071 (15)	0.0258 (15)	0.0087 (14)
C10	0.0329 (18)	0.048 (2)	0.0398 (17)	0.0071 (16)	0.0133 (14)	0.0166 (15)

### Geometric parameters (Å, °)

**Table:**

Ni1—C1	1.863 (4)	C3—H3B	0.9800
Ni1—C1^i^	1.863 (4)	C3—H3C	0.9800
Ni1—C2^i^	1.867 (3)	C4—C10^ii^	1.537 (5)
Ni1—C2	1.867 (3)	C4—C5	1.541 (4)
Ni2—N4	2.067 (3)	C5—H5A	0.9800
Ni2—N4^ii^	2.067 (3)	C5—H5B	0.9800
Ni2—N3^ii^	2.099 (3)	C5—H5C	0.9800
Ni2—N3	2.099 (3)	C6—C7	1.505 (4)
Ni2—O1^ii^	2.179 (2)	C6—H6A	0.9900
Ni2—O1	2.179 (2)	C6—H6B	0.9900
O1—H1A	0.835 (10)	C7—H7A	0.9900
O1—H1B	0.830 (10)	C7—H7B	0.9900
N1—C1	1.146 (4)	C8—C9	1.532 (4)
N2—C2	1.137 (3)	C8—H8A	0.9800
N3—C6	1.482 (4)	C8—H8B	0.9800
N3—C4	1.505 (4)	C8—H8C	0.9800
N3—H3	0.88 (3)	C9—C10	1.538 (4)
N4—C7	1.484 (4)	C9—H9	1.0000
N4—C9	1.487 (4)	C10—C4^ii^	1.537 (5)
N4—H4	0.81 (3)	C10—H10A	0.9900
C3—C4	1.528 (4)	C10—H10B	0.9900
C3—H3A	0.9800		
			
C1—Ni1—C1^i^	180.0 (2)	H3B—C3—H3C	109.5
C1—Ni1—C2^i^	88.96 (13)	N3—C4—C3	111.6 (3)
C1^i^—Ni1—C2^i^	91.04 (13)	N3—C4—C10^ii^	108.0 (2)
C1—Ni1—C2	91.04 (13)	C3—C4—C10^ii^	111.1 (3)
C1^i^—Ni1—C2	88.96 (13)	N3—C4—C5	109.2 (3)
C2^i^—Ni1—C2	180.0 (3)	C3—C4—C5	109.6 (3)
N4—Ni2—N4^ii^	180.00 (14)	C10^ii^—C4—C5	107.2 (3)
N4—Ni2—N3^ii^	94.74 (10)	C4—C5—H5A	109.5
N4^ii^—Ni2—N3^ii^	85.26 (10)	C4—C5—H5B	109.5
N4—Ni2—N3	85.26 (10)	H5A—C5—H5B	109.5
N4^ii^—Ni2—N3	94.74 (10)	C4—C5—H5C	109.5
N3^ii^—Ni2—N3	180.0	H5A—C5—H5C	109.5
N4—Ni2—O1^ii^	90.18 (10)	H5B—C5—H5C	109.5
N4^ii^—Ni2—O1^ii^	89.82 (10)	N3—C6—C7	109.3 (2)
N3^ii^—Ni2—O1^ii^	87.30 (10)	N3—C6—H6A	109.8
N3—Ni2—O1^ii^	92.70 (10)	C7—C6—H6A	109.8
N4—Ni2—O1	89.82 (10)	N3—C6—H6B	109.8
N4^ii^—Ni2—O1	90.18 (10)	C7—C6—H6B	109.8
N3^ii^—Ni2—O1	92.70 (10)	H6A—C6—H6B	108.3
N3—Ni2—O1	87.30 (10)	N4—C7—C6	109.2 (2)
O1^ii^—Ni2—O1	180.0	N4—C7—H7A	109.8
Ni2—O1—H1A	112 (2)	C6—C7—H7A	109.8
Ni2—O1—H1B	116 (2)	N4—C7—H7B	109.8
H1A—O1—H1B	98 (3)	C6—C7—H7B	109.8
C6—N3—C4	116.5 (2)	H7A—C7—H7B	108.3
C6—N3—Ni2	105.14 (17)	C9—C8—H8A	109.5
C4—N3—Ni2	122.34 (18)	C9—C8—H8B	109.5
C6—N3—H3	105.1 (19)	H8A—C8—H8B	109.5
C4—N3—H3	106 (2)	C9—C8—H8C	109.5
Ni2—N3—H3	99 (2)	H8A—C8—H8C	109.5
C7—N4—C9	115.1 (2)	H8B—C8—H8C	109.5
C7—N4—Ni2	105.86 (18)	N4—C9—C8	111.8 (3)
C9—N4—Ni2	115.77 (19)	N4—C9—C10	109.4 (2)
C7—N4—H4	105 (2)	C8—C9—C10	110.1 (3)
C9—N4—H4	107 (2)	N4—C9—H9	108.5
Ni2—N4—H4	107 (2)	C8—C9—H9	108.5
N1—C1—Ni1	177.2 (3)	C10—C9—H9	108.5
N2—C2—Ni1	178.0 (3)	C4^ii^—C10—C9	120.0 (2)
C4—C3—H3A	109.5	C4^ii^—C10—H10A	107.3
C4—C3—H3B	109.5	C9—C10—H10A	107.3
H3A—C3—H3B	109.5	C4^ii^—C10—H10B	107.3
C4—C3—H3C	109.5	C9—C10—H10B	107.3
H3A—C3—H3C	109.5	H10A—C10—H10B	106.9

Symmetry codes: (i) −*x*, −*y*, −*z*; (ii) −*x*, −*y*, −*z*+1.

### Hydrogen-bond geometry (Å, °)

**Table:**

*D*—H···*A*	*D*—H	H···*A*	*D*···*A*	*D*—H···*A*
N4—H4···N1^iii^	0.81 (3)	2.46 (3)	3.250 (4)	164 (3)
N3—H3···N2	0.88 (3)	2.34 (3)	3.201 (4)	167 (3)
O1—H1B···N2	0.83 (1)	1.96 (1)	2.789 (4)	172 (3)
O1—H1A···N1^iv^	0.84 (1)	1.94 (1)	2.775 (4)	179 (3)

Symmetry codes: (iii) *x*, −*y*−1/2, *z*+1/2; (iv) −*x*, *y*+1/2, −*z*+1/2.
